# Molecular responses to salinity stress in *Salix matsudana* (Koidz) females and males

**DOI:** 10.3389/fpls.2023.1122197

**Published:** 2023-01-27

**Authors:** Guoyuan Liu, Yuqing Wang, Bolin Lian, Ziqi Ma, Xiaoting Xiang, Jing Wu, Chunying Luo, Duojin Ma, Yanhong Chen, Chunmei Yu, Fei Zhong, Hui Wei, Jian Zhang

**Affiliations:** ^1^ School of Life Science, Nantong University, Nantong, China; ^2^ Key Lab of Landscape Plant Genetics and Breeding, Nantong, China

**Keywords:** *Salix matsudana*, salinity stress, sexual dimorphism, molecular response, RNA-Seq

## Abstract

Sexual dimorphism has commonly been found in many species. The phenotypes of *Salix matsudana* females and males are different under salinity stress. An F_1_ population was selected to compare the differences between males and females. As a result, males showed stronger roots and heavier dry weights than females. The unique molecular mechanisms of males and females under salinity stress were further analyzed based on the root transcriptome of males and females. Both males and females up-regulated systemic acquired resistance genes, such as *ADH* and oxygenase-related genes, to resist salt. Moreover, many other abiotic stress response genes were up-regulated in males to adjust to salinity stress, while females showed more down-regulation of nitrogen metabolism-related genes to decrease the harm from salinity stress. The research on salinity tolerance in *Salix matsudana* males and females would help to further understand sexual dimorphism under selection pressure and provide benefits to the ecological environment.

## Introduction

Plants have developed unique sex types to adjust to different selection pressures (Mendez and Karlsson, 2005; Olas et al., 2019). Dioecious plants have much stronger heterosis and evolutionary advantages and are more common in woody species (Barrett, 2010). Previous studies have shown that the different reproductive costs of the different sexes in dioecious plants may lead to different evolutionary directions. These evolutionary differences often lead to obvious sex differences in morphological, physiological, and ecological indicators (mainly in morphological growth, gas exchange, water use and hormone levels) and life history characteristics (Stromme et al., 2018). Sexual dimorphism has been found in *Acer tegmentosum* (Zhang et al., 2014), *Taxus wallichiana* (Zhang et al., 2009), *Taxus cuspidata* (Cedro and Iszkuło, 2011), *Populus davidiana* (Sakai and Burris, 1985) and other trees. There are significant differences between the sexes in individual survival rate, individual morphology (reproductive organs, vegetative organs, individual size, etc.), and physiological characteristics (flowering time, flowering frequency, photosynthetic characteristics, etc.).

Woody plants have to experience various abiotic stresses due to their immobility and perennial characteristics (Han et al., 2022). Sexual dimorphism is also reflected in the response to abiotic stresses (Stromme et al., 2018). Under different external conditions, plants of different sexes will appropriately adjust the allocation of resources to promote their own better development (Tonnabel et al., 2017). Previous studies have found that dioecious plants show significant sexual dimorphism in response to salt stress, drought stress, heavy metal stress, high temperature stress and low temperature stress. *Populus catharensis* (Chen et al., 2011) and *Populus yunnanensis* (Jiang et al., 2012) showed higher osmotic regulation capacity, water use efficiency, and antioxidant enzyme activity to adapt to salt stress, *Populus euphratica* males exhibit stronger drought and salt stress resistance than females (Yu et al., 2023). However, female *Populus davidianas* exhibited a taller height and more biomass accumulation than males during salt stress (Li et al., 2016). The photosynthetic rate, water use efficiency, and antioxidant enzyme activity of female *Ginkgo* were higher than those of male plants under salt stress, showing stronger salt tolerance (Zhou et al., 2018). Male *Populus yunnanensis* were more tolerant to cadmium, zinc, and lead stresses (Han et al., 2013). Male *Populus cathayana* showed an advantage under phosphorus deficiency, while female *Populus cathayana* showed an advantage under high phosphorus supply (Xia et al., 2020).

*Salix matsudana* Koidz is a typical dioecious tree species that has the characteristics of a wide distribution, strong adaptability, a short flowering cycle and fast growth and reproduction (Zhang et al., 2020). It has been widely planted in coastal beaches, riverbanks, mountains, and desert shelterbelts. Moreover, *Salix matsudana* has strong salt tolerance, which is of great significance for improving the ecological environment of saline-alkali land (Liu et al., 2021b). In this research, an F_1_ population of *Salix matsudana* was chosen to compare the salinity tolerance between *Salix matsudana* males and females. Moreover, the molecular mechanism of salinity stress in males and females was further discovered. Research on salinity tolerance in *Salix matsudana* males and females would help to further understand sexual dimorphism under selection pressure and benefit the ecological environment.

## Materials and methods

### Plant materials and tissue collection

An F_1_ individuals of *Salix matsudana* Koidz was produced by cross-breeding the male parent “9901” and the female parent “Yanjiang” in 2014 (Liu et al., 2021c). The branches of 30 female F_1_ progenies and 30 male F_1_ progenies were clipped at 10 cm lengths and 1 cm thicknesses and hydroponically cultured in water under two conditions, with water only (i.e., the normal condition, marked as “CK”) and with 0.5% NaCl solution (g/v) (i.e., the salinity stress condition, marked as “T”). The clipped branches were grown under each condition in three biological replications for RNA-seq at Nantong University in March 2022. After 30 days, the newly sprouted roots and shoots were collected from each replicate. The excised roots were immediately frozen in liquid nitrogen and stored at -80°C until use (Liu et al., 2021a).

### RNA sequencing and library construction

The excised roots were ground in liquid nitrogen. The Plant RNA Reagent Kit (Tiangen, China) was used to extract total RNA from three replications. A Nanodrop ND 2000 spectrophotometer (NanoDrop, Thermo, Waltham, MA, USA) was then used to quantify the RNAs. The RNAs were then stored at -80°C before performing RNA sequencing. Finally, Illumina sequencing technology (Illumina, San Diego, CA, USA) was employed to perform RNA sequencing by Biomarker (Beijing, China) (Chen et al., 2020).

### Analysis of sequencing data

The transcriptome reads were processed into clean, full-length reads by removing the low-quality and adapter reads (Chen et al., 2022). The assembled *Salix matsudana* Koidz. (“Yanjiang”) genome sequence was selected as the reference for paired-end read mapping (Zhang et al., 2020). The clean reads were aligned to genes of the reference genome using HiSAT2 software with default parameters (Kim et al., 2019). Then, StringTie2 was used to detect new transcripts (Pertea et al., 2015). RSEM was chosen to calculate the fragments per kilobase transcriptome per million mapped reads (FPKM) by normalizing to the length of the gene and to the number of mapped reads. To identify the differentially expressed genes (DEGs), DESeq2 was selected. Two standards were used to detect the DEGs: (1) the fold change should be no less than 2 between different libraries, and (2) the adjusted false discovery rate (FDR) should be less than 0.05 (Chen et al., 2020).

The identified genes were annotated by using the BLASTx search in the NCBI nonredundant protein database. Then, Gene Ontology (GO) categorization, clusters of eukaryotic orthologous groups (KOG), Cluster of Orthologous Groups of proteins (COG), and Kyoto Encyclopedia of Genes and Genomes (KEGG) pathway analysis were performed using BMKCloud (www.biocloud.net, version 2.0) (Liu et al., 2021a). The data that supported the findings of this study were deposited into the CNGB Sequence Archive (CNSA) of the China National GeneBank Database (CNGBdb) with accession number CNP0003818.

### Measurement of male and female phenotypes

The root depth, root width, and root cap area of 30 male and 30 female progenies under normal and saline conditions in three biological replications were measured by using Win RHIZO TRON (Xu et al., 2020). Then, all seedlings were harvested and oven-dried to measure the biomass of the whole plant by using the student t test.

## Results

### Males of *Salix matsudana* have deeper and wider roots than females

**Figure 1:**
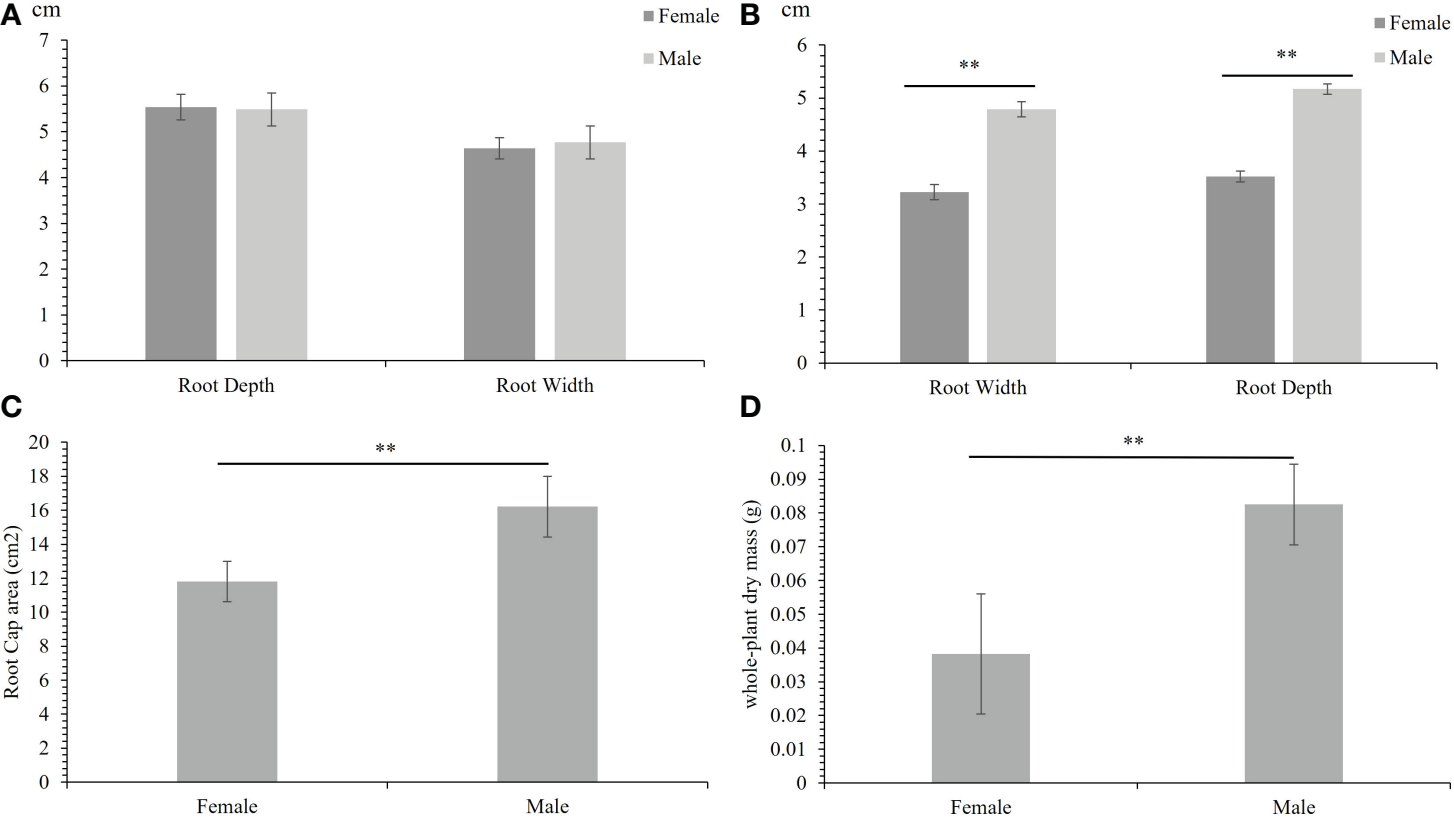
Females and males showed different responses to salinity stress. **(A)** The root depth and width of females and males under normal condition. **(B)** The root depth and width of females and males under salinity stress. **(C)** The root cap area of females and males under salinity stress. **(D)** The whole-plant dry mass of females and males under salinity stress. The error bars denote the standard error (SE), The phenotypes were significantly different at **p < 0.01.

The phenotype of 30 male F_1_ progenies and 30 female F_1_ progenies under normal and salinity conditions were measured. Under normal conditions, the root depth and width of male and female plants did not differ significantly (**Figure 1A**). In contrast, under salinity stress, the roots of male and female plants subjected to intrasexual competition were significantly different. Under salinity stress, male plants showed deeper and wider roots than female plants (**Figure 1B**). The root cap area of males was also higher than that of females (**Figure 1C**). This result means that *Salix matsudana* showed sexual dimorphism under salinity stress. Unlike in *Populus deltoides* (Li et al., 2016), male *Salix matsudana* have much more developed roots than females. We then measured the whole-plant dry mass, and the average biomass accumulation of males was higher than that of females, significantly (**Figure 1D**).

### Overview of the RNA sequencing data

To characterize the role of the response of active genes to salinity stress in males and females, deep sequencing libraries were generated using total RNA extracted from roots under normal and salinity stress conditions. After trimming off the adapter sequences and removing the low-quality reads, we obtained 19,468,575–27,771,058 clean reads for the 12 libraries; these libraries had a single read length of 90 bp and a Q30 percentage (percentage of the sequences with sequencing error rates lower than 0.1%) over 90% (**Supplementary Table S1**). The clean reads were then mapped onto the reference genome of *S. matsudana* using HISAT2. In total, 44,906 (77.64% of the 57,841 gene models in the reference genome) genes were identified as being expressed in at least one library.

### Identification of differentially expressed genes that responded to salinity stress in males and females

**Figure 2:**
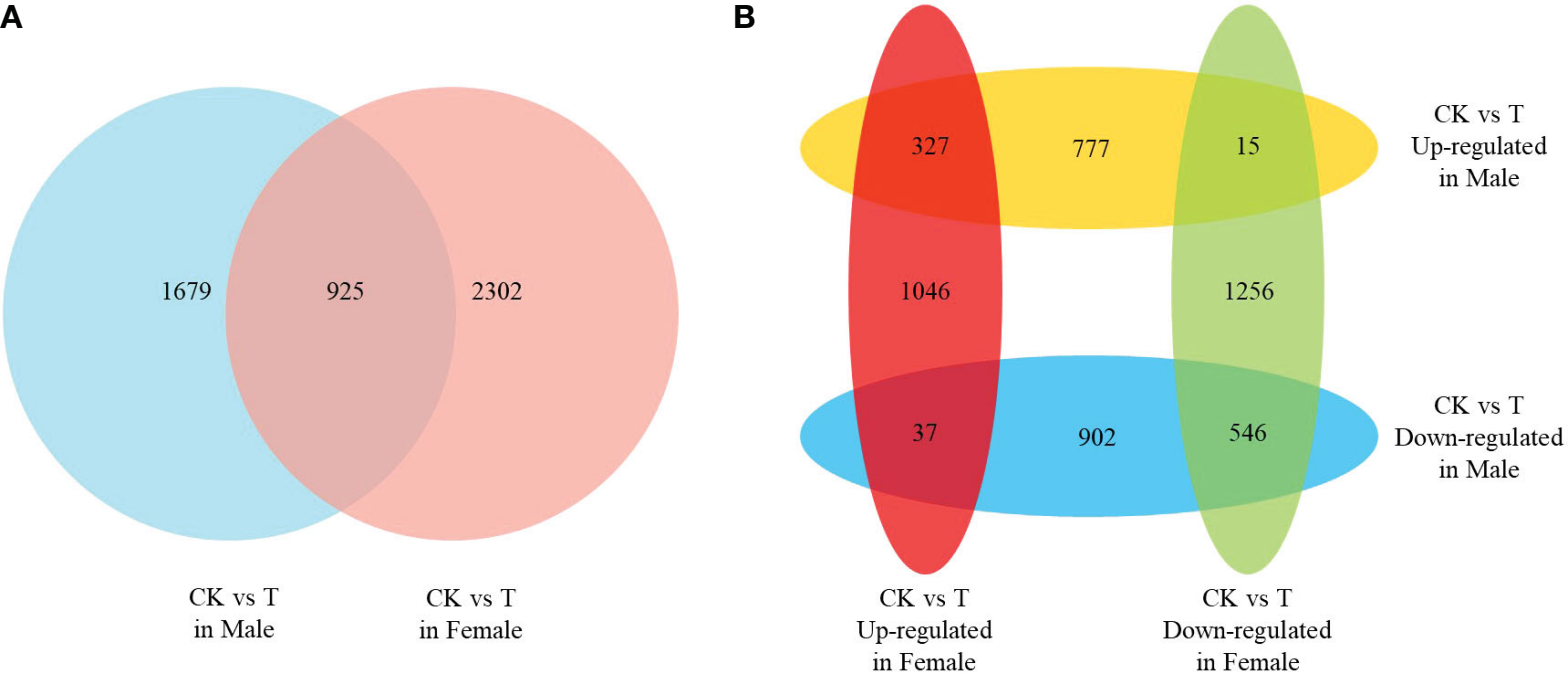
The differentially expressed genes (DEGs) of females and males response to salinity stress. **(A)** Venn analysis of the DEGs in females and males. **(B)** Venn analysis of the up- and down-regulated DEGs in females and males.

The DEGs under normal and salinity stress conditions in males and females were identified using a threshold FDR ≤ 0.05 and an absolute value of log2-fold change ≥ 1. A total of 4906 DEGs (2604 in males and 3227 in females) were identified, (**Figure 2A**). More genes were identified in the response to salinity stress in females than in males. However, only approximately one-fifth (925 in 4906) of the DEGs were identified as differentially expressed in both males and females. Compared to those under the normal condition, most genes under salinity stress conditions showed low expression (**Figure 2B**). Only 327 and 546 DEGs showed similar expression trends in males and females, respectively. Moreover, 52 DEGs were identified as showing opposite expression trends in males and females. Thirty-seven DEGs were up-regulated in females but down-regulated in males, and 15 DEGs were up-regulated in males but down-regulated in females. The results indicate that the mechanisms of the response to salinity stress in males and females may differ.

### Common protein interaction networks in response to salinity stress in males and females

**Figure 3:**
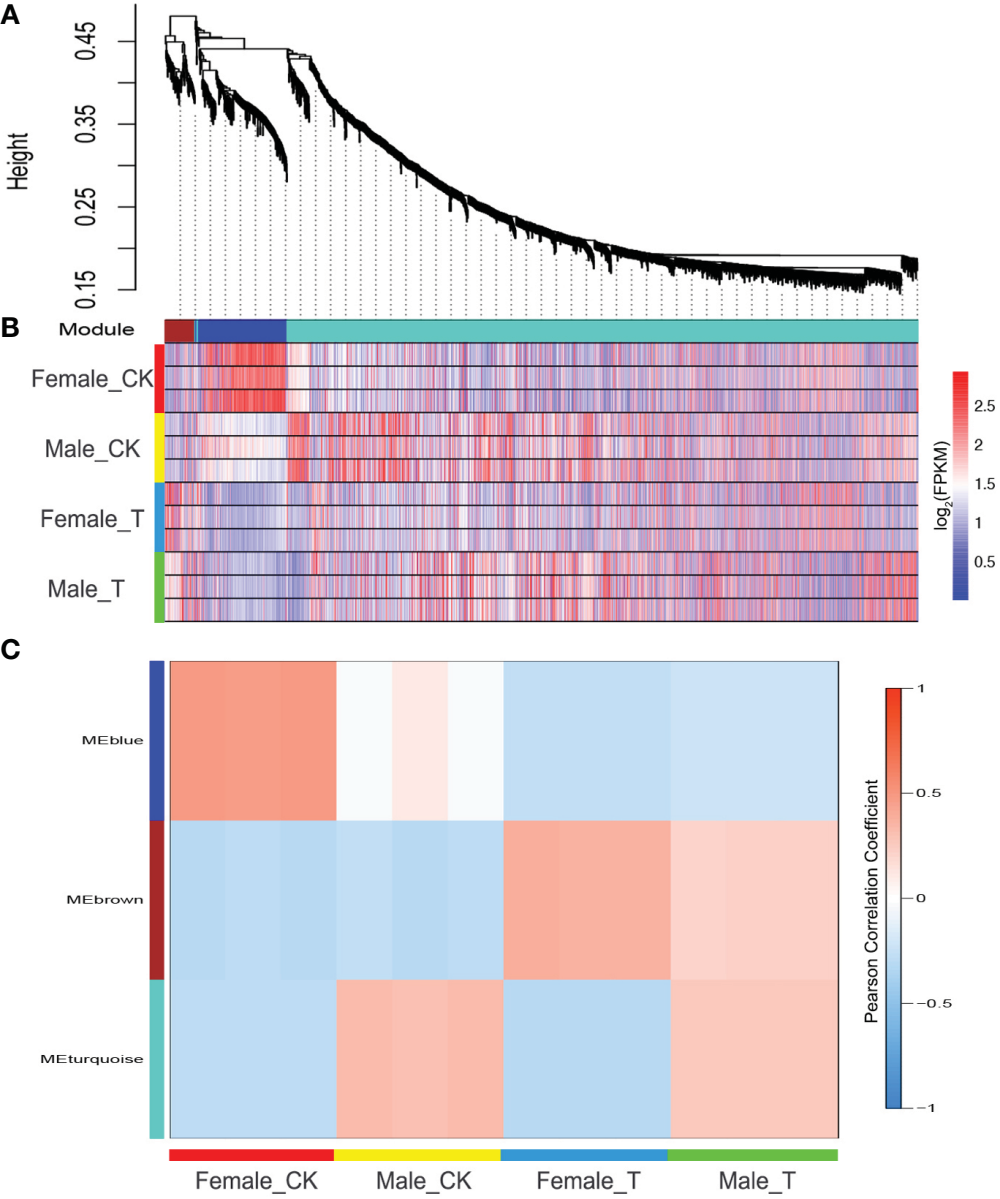
Weighted gene co-expression network analysis (WGCNA) of females and males response to salinity stress. **(A)** DEGs were classed into 3 modules. **(B)** The expression profile of DEGs in 3 modules, Blue, reduced expression; red, increased expression. **(C)** Correlation analysis of 3 modules and *Salix matsudana* under different conditions.

Weighted gene co-expression network analysis was performed to further detect common and unique salinity stress response genes in males and females. Among the 4906 DEGs, 969 could be classed into 3 modules (**Figure 3A**). In “MEbrown”, genes showed similar expression trends in males and females. These genes were up-regulated in both males and females under salinity stress, indicating that the regulation of these genes in males and females is similar (**Figure 3B**). Genes in “MEblue” showed a high correlation with female plants under normal conditions, indicating that these genes were only highly expressed in females under normal conditions and were reduced under salinity stress. In males, these genes showed similar expression levels between normal and salinity stress conditions. The genes in “MEturquoise” showed a high correlation with male plants responding to salinity stress. These genes were highly expressed in males under salinity stress and did not change much in females (**Figure 3C**).

**Figure 4:**
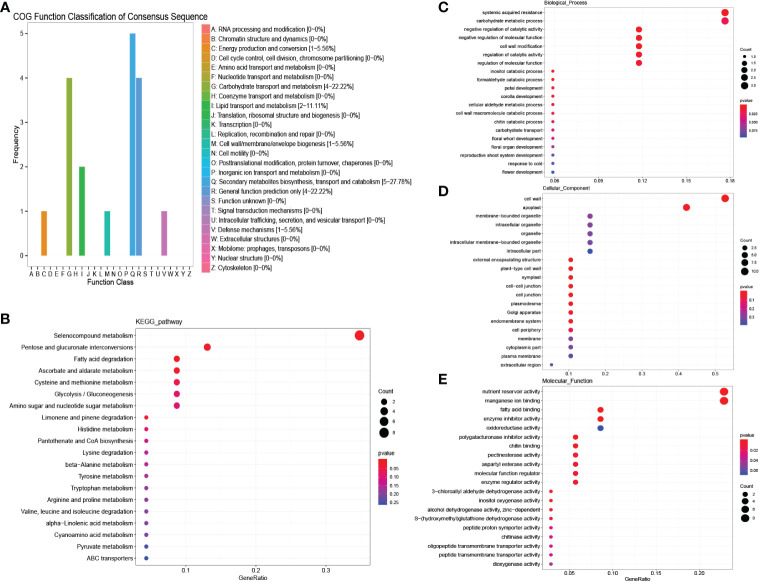
COG, KEGG and GO analysis of salinity stress responding genes in both males and females. **(A)** COG enrichment analysis of salinity stress responding genes in both males and females. **(B)** KEGG enrichment analysis of salinity stress responding genes in both males and females. **(C–E)** GO enrichment analysis of salinity stress responding genes in both males and females.

**Figure 5:**
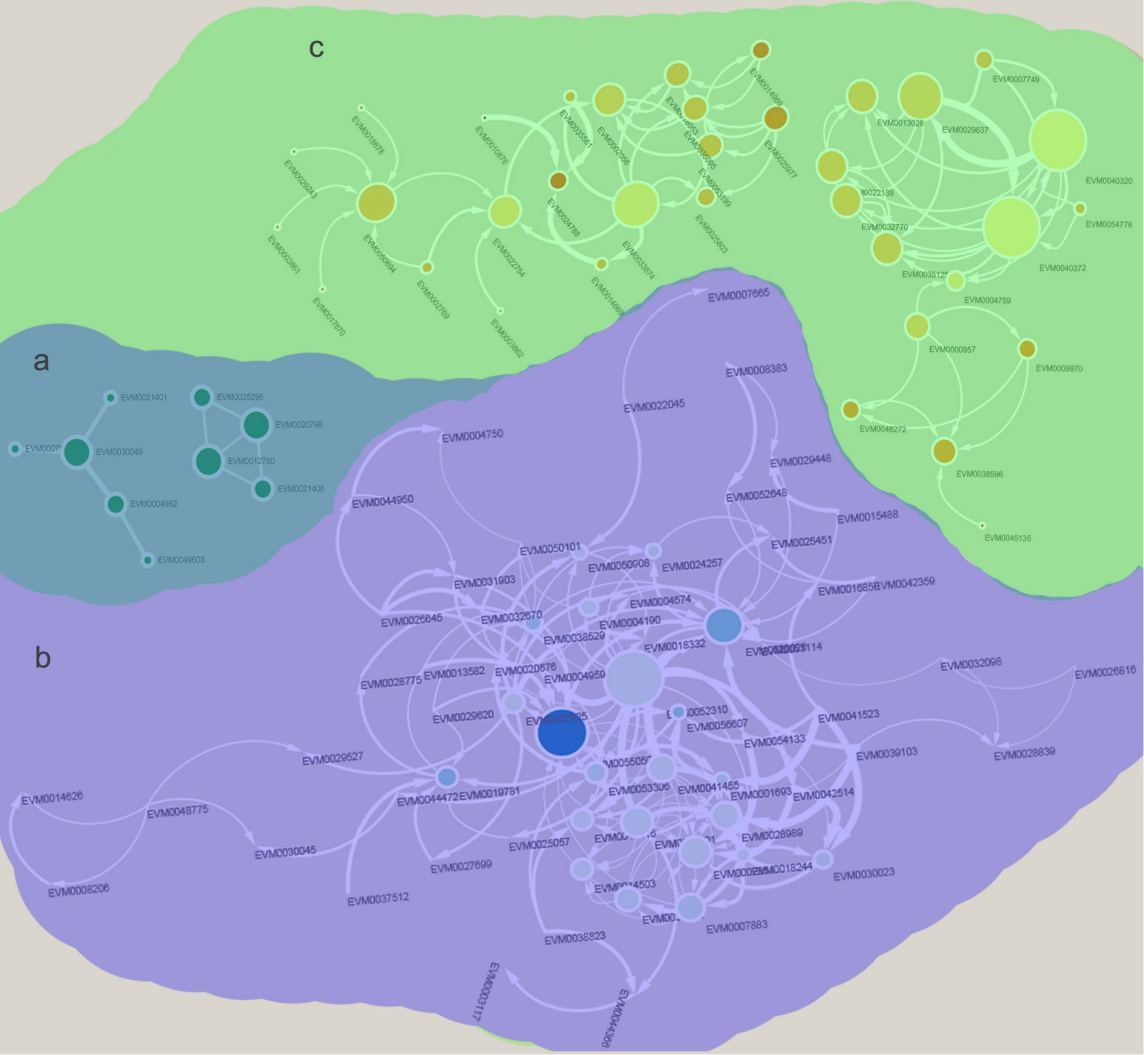
Identify of salinity stress responding protein interaction networks in *Salix matsudana* (Koidz) females and males. **(A)** Common protein interaction network in both females and males. **(B)** Male unique salinity stress responding protein interaction network. **(C)** Female unique salinity stress responding protein interaction network.

A total of 48 genes were detected in “MEbrown”, and COG enrichment analysis was performed. These genes were found to be enriched in carbohydrate transport and metabolism and secondary metabolite biosynthesis, transport and catabolism (**Figure 4A**). KEGG enrichment analysis showed that selenocompound metabolism was enriched at high levels (**Figure 4B**). GO enrichment analysis was further performed. For biological processes, the DEGs were mostly enriched in systemic acquired resistance and carbohydrate metabolic processes (**Figure 4C**). In contrast, for molecular functions, the DEGs were mostly enriched in nutrient reservoir activity (**Figure 4E**). cell wall and apoplast were detected enriched in cellular components (**Figure 4D**). According to the common salinity stress-induced protein interaction network, several genes encoding alcohol dehydrogenase (ADH) proteins, oxygenase-related genes, glycosyl hydrolase and ethylene synthesis genes were detected as hub genes (**Figure 5A**, **Table S2**). The up-regulation of these genes could help resist salinity stress (Zhao et al., 2019).

### Unique protein interaction networks in response to salinity stress in males and females

**Figure 6:**
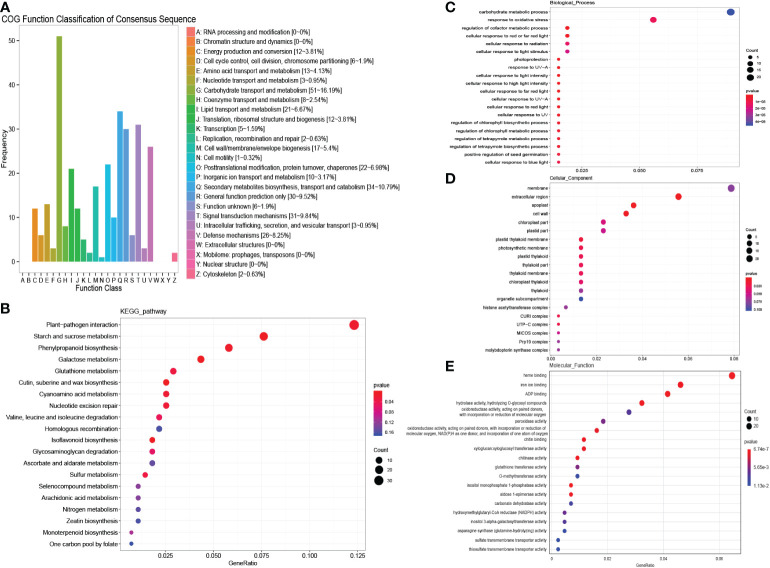
COG, KEGG and GO analysis of male unique salinity stress responding genes. **(A)** COG enrichment analysis of male unique salinity stress responding genes. **(B)** KEGG enrichment analysis of male unique salinity stress responding genes. **(C–E)** GO enrichment analysis of male unique salinity stress responding genes.

The genes responding to salinity stress that were unique to males or females were further detected. A total of 718 genes in “MEturquoise” were up-regulated in males under salinity stress. The COG enrichment analysis showed that these genes were enriched in carbohydrate transport and metabolism, secondary metabolite biosynthesis, transport and catabolism, signal transduction mechanisms and defense mechanisms (**Figure 6A**). The KEGG enrichment analysis showed that plant−pathogen interaction and starch and sucrose metabolism were significantly enriched (**Figure 6B**). According to the GO analysis, carbohydrate metabolic process and response to oxidative stress were enriched in biological processes (**Figure 6C**); membrane and extracellular region were detected in cellular components (**Figure 6D**); and many oxygen-related terms, i.e., peroxidase activity and oxidoreductase activity, were significantly enriched in molecular functions (**Figure 6E**). These results indicate that males could activate some pathways to defend against salinity stress, such as peroxidase activity, defense mechanisms, and sucrose metabolism (Jia et al., 2019). We then constructed the salinity stress-induced protein interaction network for males, and 16 hub genes were identified (**Figure 5B**, **Table S2**). These hub genes were annotated as dihydrofolate reductase (DHFR), ubiquitin, glutathione transferase (GST), and DNAJ, among others (**Table S3**). These genes were reported to reduce the damage caused by active oxygen.

**Figure 7:**
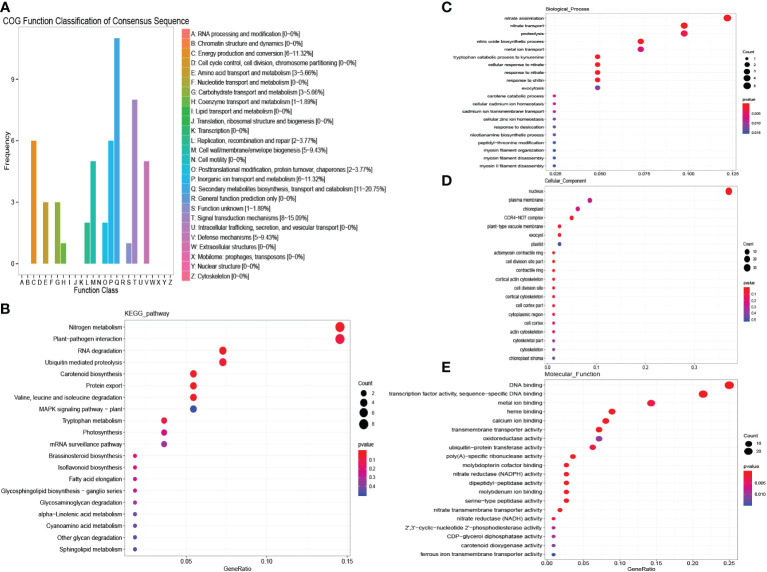
COG, KEGG and GO analysis of female unique salinity stress responding genes. **(A)** COG enrichment analysis of female unique salinity stress responding genes. **(B)** KEGG enrichment analysis of female unique salinity stress responding genes. **(C–E)** GO enrichment analysis of female unique salinity stress responding genes.

In females, 203 DEGs in “MEblue” were down-regulated under salinity stress, which means that the expression of these genes may work to the disadvantage of females when resisting salinity stress. Secondary metabolite biosynthesis, transport and catabolism, and signal transduction mechanisms were detected in the COG enrichment analysis (**Figure 7A**). In the KEGG enrichment analysis, nitrogen metabolism and plant−pathogen interaction were significantly enriched (**Figure 7B**). The GO analysis also detected several enriched nitrogen-related terms (**Figures 7C–E**). These results indicate that female plants could reduce the metabolism of nitrogen to adjust to salinity stress. These genes were then used to construct the protein interaction network of females. As a result, genes encoding nitrate reductase (NR) and nitrate transporter (NRT) were detected as hub genes (**Figure 5C**, **Table S2**). Nitrate is one of the most important sources of nitrogen in plants and can affect the growth and development of plants. Researchers have already found that NRT could regulate the distribution of lateral roots. The down-regulation of nitrate-related genes reduced the metabolism of nitrogen and plant growth. These results showed that female plants could reduce their metabolism to adjust to salinity stress.

## Discussion

### Male *Salix matsudana* plants showed higher tolerance to salinity stress than female plants

Female and male plants experience different selection pressures and have different evolutionary directions. The difference in their evolution could induce a series of morphological, physiological and ecological differences between males and females, which is called sexual dimorphism. In this research, *Salix matsudana* of both sexes were selected to identify their sexual dimorphisms under salinity stress. To suppress the interference of different genotypes, an F_1_ population was selected to perform the experiments. Males and females did not differ significantly in root depth and width under normal conditions. However, sexual dimorphism was evident under salinity stress. Male plants showed a higher tolerance to salinity stress than female plants, with deeper and wider roots and larger cap areas. Usually, males have a higher tolerance for abiotic stress because females may invest more energy into reproductive growth. Previous studies have found that under salinity stress, *Populus cathayana* and *Populus yunnanensis* males showed higher osmotic regulation ability, water use efficiency and antioxidant enzyme activity than females. This study also showed that *Salix matsudana* males gained an advantage under salinity stress. However, in *Populus deltoides* and *Ginkgo*, females are less sensitive to salinity stress than males. The results indicate that even within the same family or genus, sexual dimorphism may also differ. Interestingly, the sexual dimorphism under different abiotic stresses is not fixed. For example, male *Populus cathayana* showed an advantage under salinity (Chen et al., 2011), deficient nitrogen (Wu et al., 2021), phosphorus deficiency (Xia et al., 2020) and drought stress (Chen et al., 2014), while female *Populus cathayana* showed an advantage under high phosphorus supply (Xia et al., 2020). A probable reason for the changing sexual dimorphism could be that males and females have different selection pressures.

### Males and females have their own mechanism regarding salinity stress

To further understand the molecular response to salinity stress in *Salix matsudana* males and females, we classified the DEGs into 3 module types: male-unique modules, female-unique modules, and common modules. In the common modules, most genes were annotated into the systemic acquired resistance term. *ADH* and oxygenase-related genes were identified as hub genes. These genes were reported to regulate plant resistance to abiotic stress. *ADH* has been reported to take part in abiotic stress responses, such as those to cold stress (Davik et al., 2013; Song et al., 2017), drought stress (Senthil-Kumar et al., 2010), salinity stress (Shi et al., 2017) and flooding stress (Liu and Adams, 2007; Komatsu and Ahsan, 2009; Komatsu et al., 2012). In *Arabidopsis*, overexpression of *AtADH1* could increase the accumulation of soluble sugar and produce a stronger salt tolerance phenotype than the wild type (Shi et al., 2017).

The unique modules in males and females showed different expression trends. In males, unique response genes were up-regulated under salinity stress, while in females, most genes showed down-regulated expression trends. In males, many abiotic stress-related pathways were activated, such as starch and sucrose metabolism, peroxidase activity, and oxidoreductase activity. The hub genes, i.e., *DHFR, GST, DNAJ*, and *ubiquitin*, were annotated as being able to reduce the harm to the roots caused by oxidation. Previous studies found that *DHFR* could affect the content of chlorophyll (Van Wilder et al., 2009), and the suppression of *DHFR* could down-regulate cell proliferation and lead to cell death (Assaraf, 2007; Zheng, 2009). GST can expel oxygen free radicals from cells and reduce the damage caused by stress (Csiszar et al., 2014). In *Arabidopsis*, the expression of *GST* could increase salt tolerance and accelerate plant growth (Qi et al., 2010). In *Solanum lycopersicum*, *DNAJ* could reduce reactive oxygen species accumulation and enhance the tolerance to cold stress and heat stress (Kong et al., 2014). The overexpression of *DNAJ* could increase the tolerance to salt in *Arabidopsis* (Bekh-Ochir et al., 2013). In tobacco, the overexpression of *ubiquitin* could improve the resistance to cold, high salt and drought (Guo et al., 2008).

In contrast, unique genes in females were down-regulated under salinity stress. Most of these genes were enriched in nitrogen metabolism-related pathways. The hub genes were also annotated to encode nitrate reductase (NR) and nitrate transporter (NRT). The low expression of nitrogen metabolism-related genes could decrease the cell viability and growth of plants (Tabata et al., 2014). Previous studies also found that nitrate-related proteins could be involved in the plant response to salt stress. Under salt stress, the expression of *NRT1.5* decreased to reduce the transport of NO_3_^-^ to the bud and prevent harmful Na^+^ from entering the bud and causing injury to plants (Lin et al., 2008). By comparing the unique modules in males and females, we could develop a hypothesis: males preferred to activate abiotic stress response genes to adjust to salinity stress, while females preferred to reduce their basic nitrogen metabolism and regulate the transport of NO_3_^-^ to decrease the harm caused by salinity stress. This hypothesis could explain the phenomenon of male *Salix matsudana* plants showing higher tolerance to salinity stress than female plants.

## Conclusion

In this study, the sexual dimorphism of *Salix matsudana* under salinity stress was compared. Males showed stronger roots and heavier dry weights than females. The molecular mechanisms of males and females under salinity stress were further analyzed. As a result, both males and females upregulated systemic acquired resistance genes, such as *ADH* and oxygenase-related genes, to resist salt. Moreover, many abiotic stress response genes were up-regulated in males to adjust to salinity stress, while females preferred to down-regulate nitrogen metabolism-related genes to decrease the harm caused by salinity stress. The research on salinity tolerance in *Salix matsudana* males and females would help to further understand sexual dimorphism under selection pressure and provide benefits to the ecological environment.

## Data availability statement

The data presented in the study are deposited in the CNGB Sequence Archive (CNSA) of the China National GeneBank Database (CNGBdb) repository, accession number CNP0003818.

## Author contributions

JZ and GL conceived and designed the experiments. GL, YW, and BL performed the experiments. GL and YW analyzed the data. GL and BL wrote the manuscript. All authors contributed to the article and approved the submitted version.
